# Error-Constrained Entropy-Minimizing Strategies for Multi-UAV Deception Against Networked Radars

**DOI:** 10.3390/e27060653

**Published:** 2025-06-18

**Authors:** Honghui Ban, Jifei Pan, Zheng Wang, Rui Cui, Yuting Ming, Qiuxi Jiang

**Affiliations:** College of Electronic Engineering, National University of Defense Technology, Hefei 230037, China; banhonghui19@nudt.edu.cn (H.B.); 13865964598@139.com (Z.W.); cuirui20250605@163.com (R.C.); mingyuting23@nudt.edu.cn (Y.M.); jsc2013@sina.cn (Q.J.)

**Keywords:** compensation strategy, UAV swarm, networked radar, deception jamming, entropy minimization

## Abstract

In complex electromagnetic environments, spatial coupling uncertainties—position errors and timing jitter—increase false target information entropy, reducing strategy effectiveness and posing challenges for robust UAV swarm track deception. This paper proposes an error-constrained entropy-minimizing compensation framework to model radar/UAV errors and their spatial coupling. The framework establishes closed-form gate association conditions based on the principle of entropy minimization, ensuring mutual consistency of false target measurements across multiple radars. Two strategies are proposed to reduce false target information entropy: 1. Zonal track compensation forms dense “information entropy bands” around each preset false target by inserting auxiliary deception echoes, enhancing mutual information concentration in the measurement space; 2. Formation jamming compensation adaptively reshapes the UAV swarm into regular polygons, leveraging geometric symmetry to suppress spatial diffusion of position errors. Simulation results show that compared with traditional methods, the proposed approach reduces the spatial inconsistency entropy by 50%, improving false target consistency and radar deception reliability.

## 1. Introduction

UAV swarm has the advantages of high mobility, strong robustness, and cost-effectiveness, and the deep cooperation of multiple UAVs can realize the nonlinear growth of individual performance, which has been widely used in many fields. At the same time, advances in radar systems have significantly improved their anti-jamming capabilities, especially with the popularity of networked radar systems; a single electronic jamming platform becomes less and less capable of jamming the target radar network. Therefore, more and more scholars have begun to study the use of UAV groups to interfere with network radar, and how to use UAV groups to make network radar generate realistic phantom tracks has become a current research hot spot.

In the field of jamming technology, the concept of range deception ghost track generation proposed in 2004 initiated systematic research [1]. Scholars established the mathematical mapping relationship between flight paths and false tracks by constructing the ECAV collaborative trajectory model, laying the theoretical foundation for multi-platform cooperative deception [2,3,4,5]. As research focus shifted toward optimal control problems [6,7], Yunjun Xu proposed a method for designing real-time feasible and optimal trajectories inspired by motion camouflage, reducing problem dimensionality and improving computational efficiency [8]. This work provided critical theoretical support for subsequent technological advancements. Zhiping Ouyang developed a multi-UAV cooperative track deception optimization model, analyzing UAV positions and motion states to derive motion parameters through simulation and determining the minimum number of UAVs and optimal spacing required for effective radar network jamming [9]; N. Dhananjay addressed the generation of coherent virtual trajectories using ECAVs to deceive radar networks, providing sufficient conditions for feasible ECAV trajectories under proportional navigation missile-like scenarios. Line-of-sight guidance laws were employed to control ECAVs, and performance metrics were proposed to evaluate trajectory coherence [10]. Zhe Fan investigated deception rules for UAV swarms attacking radar networks, establishing a geometric model to minimize the number of UAVs required for false trajectory generation and offering insights into enhancing swarm electronic countermeasures [11]. In terms of application scenario expansion, Ratnoo constructed an adaptable deception formation controller for arbitrary initial positions based on the LOSG principle [12]; Zhang, Y. innovatively proposed a closed-form solution method for dual moving-source jamming against monopulse radars [13]; and Muhammad Abubakar Ali targeted the Linear Frequency Modulation (LFM) pulse Doppler radar. By intercepting the signal parameters and adding positive and negative delays, it was verified through simulation that highly similar false targets could be formed before and after the real echoes, confusing the radar discrimination [14]; and Bin Rao proposed a cooperative deception method based on noncoherent dual-source jamming for distributed radar fusion systems. By statistically controlling the amplitude ratio and time delay of jamming signals, noncoherent angle scintillation effects are induced between decoy signals and target echoes. This causes radar angle tracking to deviate from the true target, undermining the ‘common origin’ feature of targets and degrading trajectory fusion discriminability. Simulation results validate the feasibility of this method [15]. Yubing Wang analyzed the impact of radar positioning error and UAV jitter error on track spoofing in scenarios with missing prior information [16]. Pan Yu reviewed the development process of interference signal design, proposed a general formula for unified interference waveform design, pointed out the existing problems of the current interference from the signal level, and proposed improvement directions such as compound interference and the introduction of machine learning algorithms [17].

Radar anti-jamming technologies have undergone iterative upgrades, with multidimensional innovations enhancing system stability. Zhao et al. [18] exploited spatial scattering differences and multi-channel correlation tests to detect false targets under arbitrary deception. Li [19] employed Hermitian distance for true/false discrimination in distributed radars. Qiang Li [20] effectively identifies false targets in the scenario of combined distance-speed deception by utilizing the difference in the statistical distribution of velocities between real targets and false targets. Zhe Ji [21] proposes a radar networking anti-multi-range false target interference technology based on azimuth measurement correlation and nearest neighbor correlation. Hengli Yu [22] utilizes the spatial coherence difference between the target echo and the interference signal in multi-station radar and improves the target detection performance by combining noise subspace projection with generalized likelihood ratio detection (GLRT). Yang [23] addressed false data injection attacks with a confidence covariance intersection algorithm that adaptively down-weights suspect data. Zhou [24] fused bispectral features via Bayesian decision theory and kernel density estimation to distinguish true echoes, deception jamming, and noise. Zhang [25] proposed a non-coherent integration detection algorithm for joint jamming suppression and target detection. Han [26] introduced an active/passive networked radar method leveraging radar position error during initialization and sequential track correlation for false target elimination. Yang [27] fused nearest-neighbor algorithms with passive radar to improve tracking accuracy, and Zhang [28] used multi-perspective fully polarimetric observations in a multiple hypothesis testing model. Cheng Feng [29] proposed an optimization method for radar anti-jamming strategies based on key time nodes. The behavior of the interfering party was modeled through the Markov decision process to enhance the concealment of the strategy.

In recent years, the intelligent evolution of electronic warfare has accelerated. Li introduced a multi-agent deep-reinforcement-learning motion-allocation algorithm that adaptively re-positions UAV swarms, markedly enhancing cooperative jamming [30]; Yang refined MADDPG with clipped double Q-networks and delayed policy updates, curbing over-estimation and boosting pursuit efficiency [31]; Guo broke the SAR deception–template bottleneck via OTSCycGAN [32]; Haowei Zhang et al. addressed the resource allocation problem in UAV-aided joint radar and communication (JRC) networks. They proposed the JCAPASA and JPBSA strategies, taking the Cramér-Rao Lower Bound (CRLB) as the optimization metric to jointly optimize resource allocation, including power and subchannels [33,34]; Sun enhanced distributed-radar tracking with the ADJ-JRAPS joint strategy [35]; Zhang realized simultaneous gains in transmission efficiency and detection through fusion-based techniques [36]; Yongkang Wang utilized the integration of multiple networks to extract the spatiotemporal characteristics of trajectories and effectively identify active deception interference [37]; XiYu combined with SVM proposed a multi-domain feature fusion method based on time-domain envelope fluctuations and frequency-domain similarity for interference identification [38]; and Tan’s PASS-Net excelled in few-shot modulation classification [39]. Collectively, these studies herald a shift toward intelligent, collaborative, cross-domain jamming. The main contributions of this paper are:

- Most cooperative-deception studies presuppose error-free conditions. We separately model radar prior-position, UAV-position. and time-delay errors, then integrate them into a spatially coupled comprehensive model. Using the principle of homologous testing, we derive conditions under which false targets satisfy spatial-consistency checks throughout track formation.
- To offset real-world errors and raise deception success, we propose two compensation schemes: (1) Zonal Track Compensation, which algebraically quantifies offsets under coupled errors; and (2) UAV Formation Compensation, which geometrically suppresses horizontal UAV position errors.
- Numerical simulations show that while individual errors displace false targets, the proposed schemes shrink the required range gate and, when combined, deliver the best performance—confirming their practicality and effectiveness.

## 2. Related Work

### 2.1. Cooperative Track Deception

**Figure 1:**
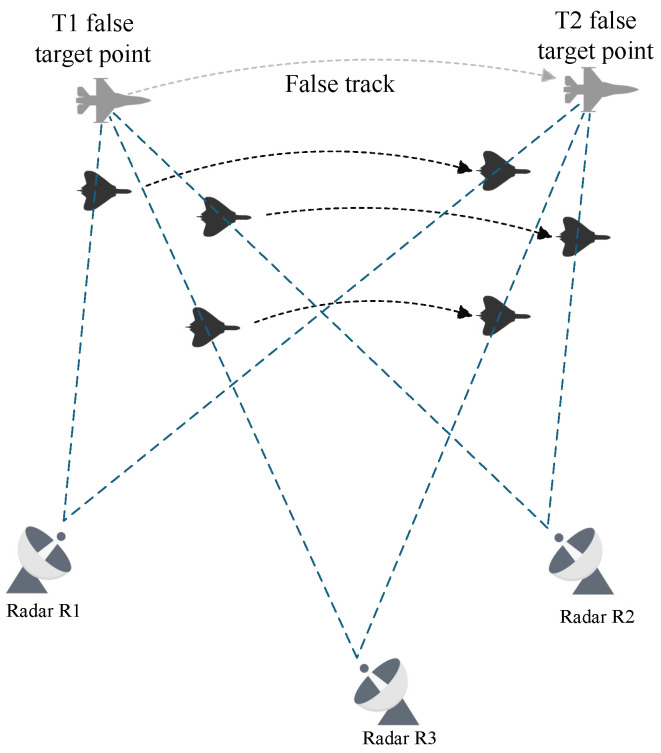
Multi-UAV Cooperative Track Deception Against Networked Radars.

Track deception jamming, a cornerstone of radar countermeasures, is broadly classified into two paradigms: repeater-based and generative. This study focuses on the mechanistic analysis of repeater-based track deception, where the core lies in precise time-frequency modulation of intercepted radar signals by digital radio frequency memory (DRFM) systems mounted on unmanned aerial vehicles (UAVs). Through a controllable delay (or pre-advancement) retransmission mechanism, range deception echoes are synthesized at the radar receiver frontend. These echoes induce the radar to interpret false signals as target position offsets (either farther or closer), culminating in the formation of deceptive track sequences [40]. In the netted radar engagement model depicted in Figure 1, three UAVs operate in a clustered formation to implement jamming. During each radar scan cycle, the jamming cluster employs mainlobe directional jamming to exploit geometric positioning relationships for constructing false track points in the radar’s observation space: each cycle’s false target is positioned along the extension of the line connecting the radar and the UAV [10]. Following “consilience criterion” verification and multi-radar data fusion, these discrete false targets are processed into spatiotemporally coherent false tracks, thereby achieving effective deception of the radar tracking system.

In this scenario, the basic assumptions for implementing track deception jamming are as follows.

1. The UAVs possess excellent stealth performance and high maneuverability.
2. A priori information such as the spatial positions of the target radars is available.
3. The jammer has the capability of high-precision signal replication.

### 2.2. Mathematical Model of Track Deception

**Figure 2:**
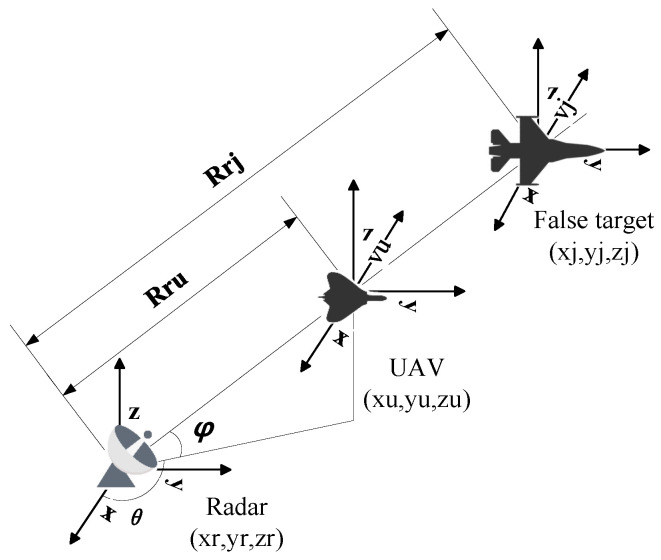
Basic Model of Track Deception.

Before using UAV swarm to implement track deception, we established a mathematical model with a single UAV jamming a single radar, as shown in Figure 2. Assume that the coordinates of the radar, UAV, and false target are $\left(x_{r},y_{r},z_{r}\right)$, $\left(x_{u},y_{u},z_{u}\right)$, and $\left(x_{j},y_{j},z_{j}\right)$, respectively, and the distance from the UAV to the radar is $R_{ru}$, the azimuth Angle is φ, the pitch Angle is θ, the distance from the false target to the radar is $R_{rj}$, the velocity of the UAV is $v_{u}$, and the velocity of the false target is $v_{j}$.

Since mainlobe jamming requires radar, UAV, and false target to be in a straight line, the following relationship is satisfied:

(1) $$\frac{x_{u}-x_{r}}{x_{j}-x_{r}}=\frac{y_{u}-y_{r}}{y_{j}-y_{r}}=\frac{z_{u}-z_{r}}{z_{j}-z_{r}}$$

In general, the coordinates of the false target and the radar are known, and the flight altitude range of the UAV is known. Assuming that the altitude of the UAV is *h*, the position of the UAV can be obtained as follows:

(2) $$\left\{\begin{array}{c} x_{u}=x_{r}+\frac{\left(h-z_{r}\right)\left(x_{j}-x_{r}\right)}{z_{j}-z_{r}}\\ y_{u}=y_{r}+\frac{\left(h-z_{r}\right)\left(x_{j}-x_{r}\right)}{z_{j}-z_{r}}\\ z_{u}=h \end{array}\right.$$

Assume that the azimuth angle of the false target velocity is α and the pitch angle is β, then the velocity in each direction is as follows:

(3) $$\left\{\begin{array}{c} \dot{x}=v_{j}\cos\beta \cos\alpha \\ \dot{y}=v_{j}\cos\beta \sin\alpha \\ \dot{z}=v_{j}\sin\beta \end{array}\right.$$

The kinematic equation of the false targets is as follows:

(4) $$\left[\begin{array}{c} \dot{r}\\ r\dot{\theta }\cos\varphi \\ r\dot{\varphi } \end{array}\right]=\left[\begin{array}{ccc} \cos\varphi \cos\theta & \cos\varphi \sin\theta & \sin\varphi \\ -\sin\theta & \cos\theta & 0\\ -\sin\varphi \cos\theta & -\sin\varphi \sin\theta & \cos\varphi \end{array}\right]\left[\begin{array}{c} \dot{x}\\ \dot{y}\\ \dot{z} \end{array}\right]$$

Substitute Equation (3) into Equation (4).

(5) $$\left[\begin{array}{c} \dot{r}\\ r\dot{\theta }\cos\varphi \\ r\dot{\varphi } \end{array}\right]=\left[\begin{array}{ccc} \cos\varphi \cos\theta & \cos\varphi \sin\theta & \sin\varphi \\ -\sin\theta & \cos\theta & 0\\ -\sin\varphi \cos\theta & -\sin\varphi \sin\theta & \cos\varphi \end{array}\right]\left[\begin{array}{c} v\cos\alpha \cos\beta \\ v\cos\alpha \sin\beta \\ v\sin\alpha \end{array}\right]$$

Thus, the system of differential equations for the position of the target in the spherical coordinate system can be solved as follows:

(6) $$\left\{\begin{array}{c} \dot{r}=v\left(\cos\varphi \cos\theta \cos\alpha \cos\beta +\cos\varphi \sin\theta \cos\alpha \sin\beta +\sin\varphi \sin\alpha \right)\\ \dot{\varphi }=\frac{v}{r\cos\varphi }\left(-\sin\theta \cos\alpha \cos\beta +\cos\theta \cos\alpha \sin\beta \right)\\ \dot{\theta }=\frac{v}{r}\left(-\sin\varphi \cos\theta \cos\alpha \cos\beta -\sin\varphi \sin\theta \cos\alpha \sin\beta +\cos\varphi \sin\alpha \right) \end{array}\right.$$

In summary, the equations of motion of the UAV are as follows:

(7) $$\left\{\begin{array}{c} v_{u}={\left({\dot{r}}_{u}^{2}+{\left(r_{u}\dot{\theta }\cos\varphi \right)}^{2}+{\left(r_{u}\dot{\varphi }\right)}^{2}\right)}^{1/2}\\ \cos\beta_{u}=\frac{r_{u}\left(\dot{\theta }\cos\varphi \sin\varphi \cos\theta -\dot{\varphi }\sin\theta \right)}{v_{u}\sin\varphi } \end{array}\right.$$

### 2.3. Conditions for Cooperative Deception Jamming Against Networked Radars

**Figure 3:**
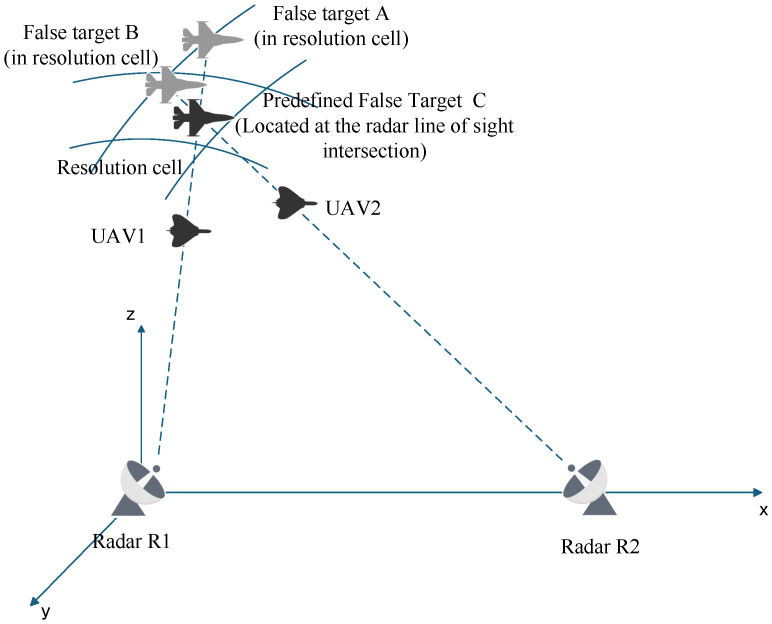
Schematic Diagram of Spatial Resolution Cell.

During practical implementation, various uncertainties cause the actual false target points to disperse [41]. As shown in Figure 3, false targets A and B do not lie within the same spatial resolution cell. Thus, it is essential to analyze errors and investigate cooperative deception strategies under error conditions.

Since mainlobe jamming requires radar, UAV, and false target to be in a straight line, the following relationship is satisfied:

Assume that at time *k*, the measurements of radar 1 and radar 2 are $\rho_{k}^{1}$ and $\rho_{k}^{2}$, respectively. When transformed into each other’s coordinate systems, these measurements yield $\rho_{k}^{12\prime }$ and $\rho_{k}^{21\prime }$, with correlation gate thresholds $\rho_{T_{1}}$ and $\rho_{T_{2}}$, respectively.

(8) $$\left\{\begin{array}{c} \left|\rho_{k}^{1}-\rho_{k}^{21\prime }\right|\leq \rho_{T_{1}}\\ \left|\rho_{k}^{2}-\rho_{k}^{12\prime }\right|\leq \rho_{T_{2}} \end{array}\right.$$

If Equation (8) is satisfied, the measurements of radar 1 and radar 2 are considered associated. If measurements from different radars do not satisfy the association condition, they fail the homologous inspection and are identified as false targets to be eliminated. This situation results in missing track points on false tracks or even in the inability to generate valid tracks. All subsequent analyses in this paper are based on this principle.

In the track deception scenario for networked radars, the deception effectiveness of false targets highly depends on the degree of consistency between their spatial states and the predefined deception strategy. To precisely quantify the uncertainty exhibited by false targets during dynamic interaction processes, this paper constructs a mathematical expression framework for information entropy from the perspective of the Euclidean distance of spatial position deviations of false targets. The specific expression of information entropy for false targets is given as follows:

(9) $$H=-\sum_{j=1}^{K}p_{j}\log_{2}p_{j}$$

where $p_{j}$ denotes the probability that the false target generated by the *j*-th UAV has a distance smaller than the threshold from the predefined false target. A higher entropy value indicates a larger Euclidean distance deviation of the generated false target from the predefined one, implying a worse jamming effect.

## 3. System Model

Since the scenario in this paper is premised on main lobe deception jamming, radars, UAVs, and false targets all lie along the same line. For false targets located on the line of sight between radars and UAVs, their spatial positions are jointly determined by UAV positions, radar positions, and time delay parameters. Consequently, false targets generated by different UAVs jamming different radars within the networked radar system must coincide in position; otherwise, they will be identified as false targets and eliminated by the system.

Current solutions for track deception primarily adopt a “reverse approach,” where UAV positions at each time are derived backward based on pre-defined false tracks and target radar positions [42]. Under ideal conditions, parameters of false targets detected by individual radars are coupled across domains. However, in real-world environments, various uncertainties cause false targets to be eliminated during fusion processing. Therefore, it is essential to analyze and account for these errors and their impacts.

### 3.1. Error Analysis of Radar A Priori Position

**Figure 4:**
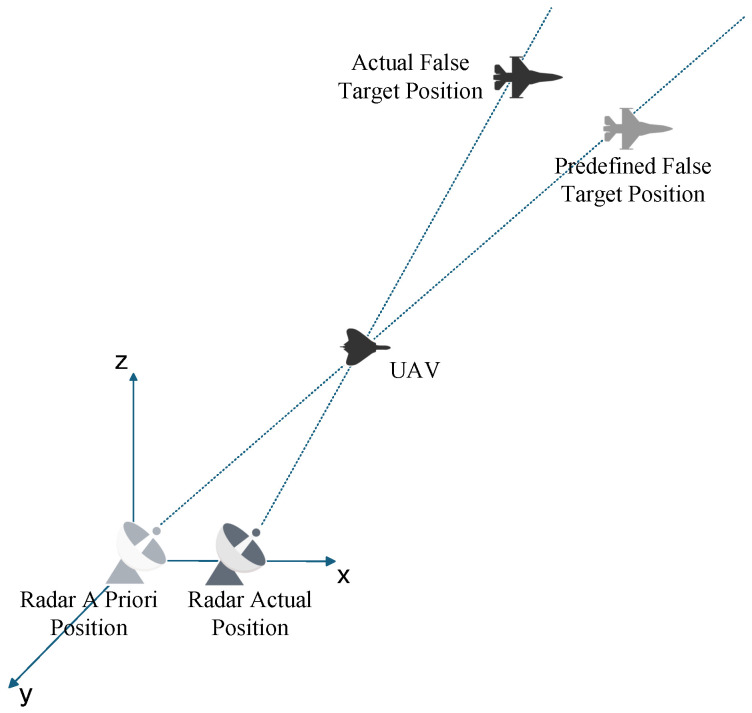
False Target offset Induced by Radar A Priori Position Errors.

The radar prior position error is mainly caused by the positioning system. In the actual positioning process, complex terrains such as mountainous areas will affect the propagation of radar signals. The signal will be occluded, reflected, or refracted by the mountain, resulting in the distortion of the positioning system signal. However, in a complex electromagnetic environment, interference signals may mislead the positioning system to make misjudgments and eventually lead to positioning deviation. In addition, the technical limitations of the positioning system itself may also make the obtained radar prior position deviate from the actual position. The implementation of main lobe track deception jamming relies on the line of sight (LOS) criterion. When false tracks and UAV positions are predetermined without offsets, discrepancies between detected and actual radar positions will cause the predefined false targets to shift, leading to the splitting of false target points detected by individual radars within the networked radar system. Figure 4 illustrates that errors between Radar A Priori Position and Radar Actual Position result in False Target offset.

Assume that the radar a priori position error magnitude is $\Delta R_{r}$, the resulting false target offset is $\Delta r_{r}$, the distance from the radar a priori position to the UAV is $R_{ru}$, and the distance from the UAV to the predefined false target is $R_{uj}$, where the ratio of $R_{ru}$ to $R_{uj}$ is *p*. Through geometric approximation, the relationship between the offset and the error can be approximated as follows.

(10) $$\Delta r_{r}=\Delta R_{r}\left(\frac{1-p}{p}\right)$$

From Figure 3 and the triangle inequality theorem, the following system of inequalities can be derived.

(11) $$\left\{\begin{array}{c} \left|\rho_{k}^{1}-\rho_{k}^{21\prime }\right|=\left|\left|{R_{1}}^{\prime }A\right|-\left|{R_{1}}^{\prime }B\right|\right|<\left|AB\right|<\left|AC\right|+\left|BC\right|=\Delta r_{1}+\Delta r_{2}\\ \left|\rho_{k}^{2}-\rho_{k}^{12\prime }\right|=\left|\left|{R_{2}}^{\prime }A\right|-\left|{R_{2}}^{\prime }B\right|\right|<\left|AB\right|<\left|AC\right|+\left|BC\right|=\Delta r_{1}+\Delta r_{2} \end{array}\right.$$

Therefore, to satisfy measurement association, it follows from Equations (8) and (11) that the following conditions must be met.

(12) $$\left\{\begin{array}{c} \Delta r_{1}+\Delta r_{2}<\rho_{T_{1}}\\ \Delta r_{1}+\Delta r_{2}<\rho_{T_{2}} \end{array}\right.$$

Assume that the magnitude of radar a priori position error is known (if only the error range is determined, the maximum value is selected). However, since the direction of the error vector is unknown, to derive conditions that ensure measurement association, it is necessary to calculate the false target distribution to determine the maximum false target deviation.

**Figure 5:**
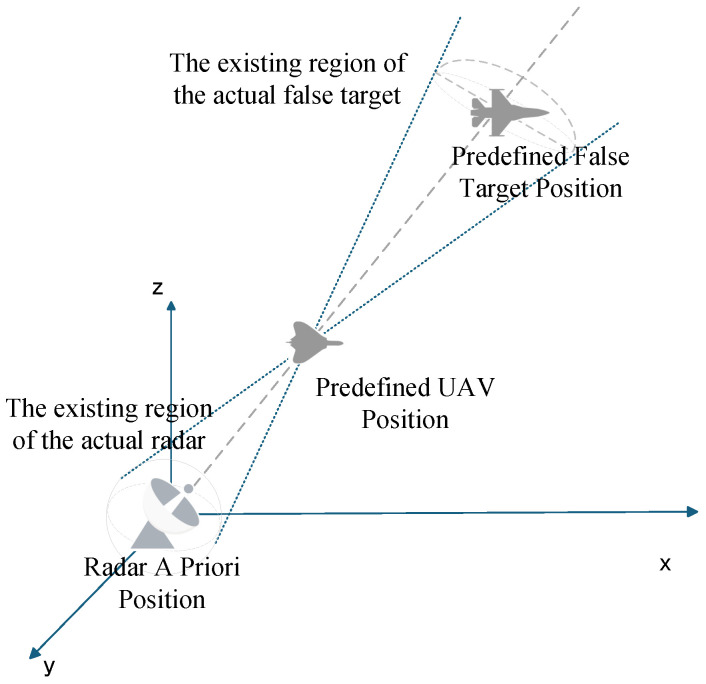
False target distribution influenced by Radar A Priori Position Errors.

Figure 5 illustrates the false target distribution influenced solely by radar prior position error. Assume that the UAV position is $\left(x_{u},y_{u},z_{u}\right)$, the actual false target position is $\left(x_{j},y_{j},z_{j}\right)$, and the deception distance is d. The distribution of actual false targets can be derived as follows:

(13) $$\left\{\begin{array}{c} {\left(x_{j}-x_{u}\right)}^{2}+{\left(y_{j}-y_{u}\right)}^{2}+{\left(z_{j}-z_{u}\right)}^{2}=d^{2}\\ x_{u}x_{j}+y_{u}y_{j}+z_{u}z_{j}\geq x_{u}^{2}+y_{u}^{2}+z_{u}^{2} \end{array}\right.$$

Based on the calculation of entropy in Equation (9), we need to obtain the probability that the deviation distance is less than a certain threshold. Therefore, through derivation, the distribution function of the threshold $\Delta r_{r,T}$ under this condition can be obtained as follows:

(14) $$F\left(\Delta r_{r,T}\right)=\left\{\begin{array}{cc} 0 & \Delta r_{r,T}<0\\ 1-\frac{\left(1-\frac{\Delta {r_{r,T}}^{2}}{2d^{2}}\right)\sqrt{\Delta R_{r}^{2}-\frac{R_{ru}^{2}\Delta {r_{r,T}}^{2}}{2d^{2}}\left(2-\frac{\Delta {r_{r,T}}^{2}}{2d^{2}}\right)}}{\Delta R_{r}} & 0\leq \Delta r_{r,T}\leq \Delta r_{r,\max}\\ 1 & \Delta r_{r,T}\geq \Delta r_{r,\max} \end{array}\right.$$

Furthermore, we can derive the maximum False Target Deviation when only radar a priori position errors exist, as follows.

(15) $$\Delta {r_{r,\max}}_{k}^{i}=d_{k}^{i}\sqrt{2-\frac{2{R_{ru,}}_{k}^{i}}{\sqrt{{\left({R_{ru,}}_{k}^{i}\right)}^{2}+{\left(\Delta {R_{r,}}_{k}^{i}\right)}^{2}}}}$$

In Equation (15), at time *k*, the maximum distance of the false target offset caused by Radar *i* a priori position error is $\Delta {r_{r,\max}}_{k}^{i}$, where the deception distance is $d_{k}^{i}$, the distance from the radar a priori position to the predefined UAV position is ${R_{ru,}}_{k}^{i}$, and the radar a priori position error is $\Delta {R_{r,}}_{k}^{i}$.

### 3.2. Error Analysis of UAV Position

**Figure 6:**
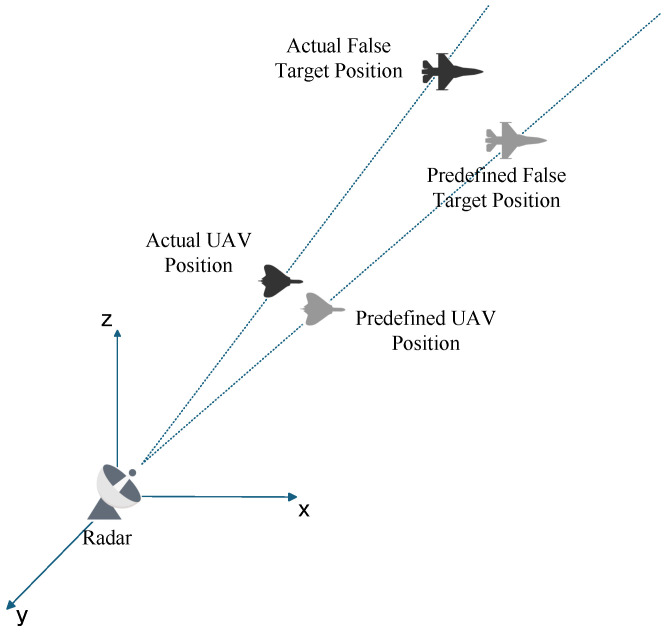
False Target offset Induced by UAV Position Errors.

The position error of a UAV is mainly caused by its own characteristics and external environmental factors. Due to the small size and light weight of the UAV, it is extremely vulnerable to airflow disturbance when flying at high altitude. In addition, the positioning system on board has real-time calculation error, and the signal is also susceptible to external interference, which will cause positioning deviation. From the perspective of the external environment, the communication delay has a particularly significant impact on the position control of UAV. There is a time lag between the command issued by the ground control station and the command received and executed by the UAV. This lag will lead to the deviation between the actual position of the UAV and the expected position in the high-speed flight state and then cause the position deviation of the false target. Figure 6 illustrates that position errors between the actual and predefined UAV positions directly result in false target misalignment.

Assume that the radar a priori position error magnitude is $\Delta R_{u}$, and the resulting false target offset is $\Delta r_{u}$. Through geometric approximation, the relationship between the offset and the error can be approximated as follows.

(16) $$\Delta r_{u}=\Delta R_{u}\left(\frac{1}{p}\right)$$

**Figure 7:**
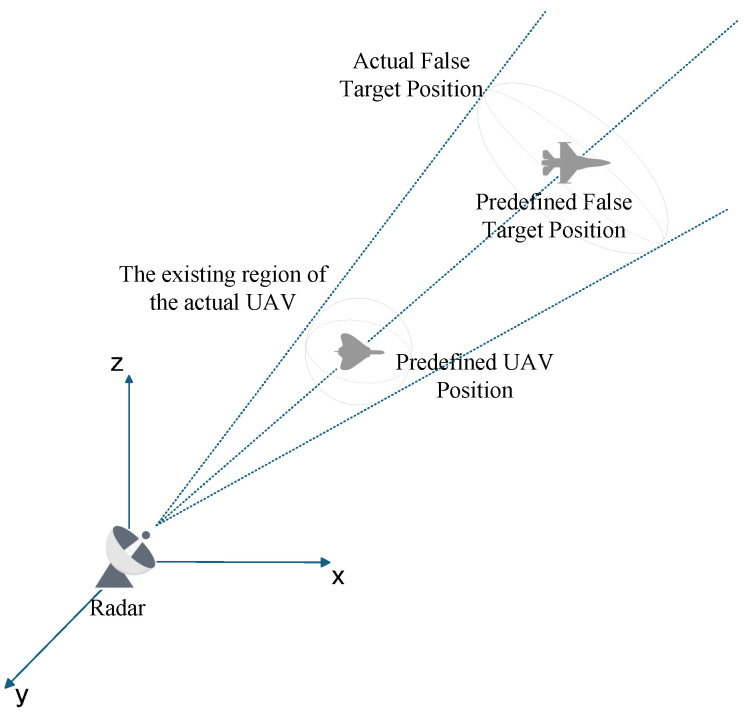
False target distribution influenced by UAV Position Errors.

Following the same methodology as the radar a priori position error analysis, we first derive the distribution of actual false target positions, then determine the maximum false target deviation, and finally derive the measurement association conditions. Figure 7 illustrates the false target distribution influenced solely by UAV position error.

Assume that the coordinates of the predefined UAV position are $\left(x_{u},y_{u},z_{u}\right)$, the spherical coordinates of the actual UAV position are $\left(\rho_{u^{\prime }},\theta_{u^{\prime }},\varphi_{u^{\prime }}\right)$, the angle between the predefined UAV position and the actual UAV position in the *z*-axis direction is $\varphi_{uu^{\prime }}$, the spherical coordinates of the actual false target position are $\left(\rho_{j},\theta_{j},\varphi_{j}\right)$, and the deception distance is *d*. Define:

$$\vec{A}=\left(x_{u},y_{u},z_{u}\right),\;\vec{B}=\left(\sin\varphi_{u^{\prime }}\cos\theta_{u^{\prime }},\sin\varphi_{u^{\prime }}\sin\theta_{u^{\prime }},\cos\varphi_{u^{\prime }}\right)$$

$$C=\Delta {r_{u}}^{2}+2d^{2}-2d\Delta r_{u}cos\varphi_{uu^{\prime }}+\frac{2d\left(\Delta {r_{u}}^{2}-dR_{ru}+\Delta r_{u}cos\varphi_{uu^{\prime }}\left(R_{ru}-d\right)\right)}{\sqrt{R_{ru}^{2}+\Delta {r_{u}}^{2}+2R_{ru}\Delta r_{u}cos\varphi_{uu^{\prime }}}}$$

The distribution of actual false targets can be derived as follows:

(17) $$\left\{\begin{array}{c} \rho_{j}=\vec{A}\cdot \vec{B}\pm \sqrt{{\left(\vec{A}\cdot \vec{B}\right)}^{2}-\left(\vec{A}\cdot \vec{A}-\Delta R_{u}^{2}\right)}+d\\ \theta_{j}=\theta_{u^{\prime }}\\ \varphi_{j}=\varphi_{u^{\prime }} \end{array}\right.$$

Similarly, we can derive the distribution function under this condition as follows:

(18) $$F\left(\Delta r_{u,T}\right)=\left\{\begin{array}{cc} 0 & \Delta r_{u,T}<\Delta R_{u}\\ \frac{1}{2}\int_{-1}^{1}1_{\left\{C\leq \Delta {r_{u,T}}^{2}\right\}}\;dcos\varphi_{uu^{\prime }} & \Delta R_{u}\leq \Delta r_{u,T}\leq \Delta r_{u,\max}\\ 1 & \Delta r_{r,T}\geq \Delta r_{u,\max} \end{array}\right.$$

We can derive the maximum False Target offset when only UAV position errors exist, as follows.

(19) $$\Delta {r_{u,\max}}_{k}^{i}=\sqrt{{\left(\Delta {R_{u,}}_{k}^{j}\right)}^{2}+2{d_{k}^{i}}^{2}+\frac{2(d_{k}^{i}{\left(\Delta {R_{u,}}_{k}^{j}\right)}^{2}-{R_{ru,}}_{k}^{i}{\left(d_{k}^{i}\right)}^{2})}{\sqrt{{\left({R_{ru,}}_{k}^{i}\right)}^{2}+{\left(\Delta {R_{u,}}_{k}^{j}\right)}^{2}}}}$$

In Equation (19), at time *k*, the maximum distance of the false target offset caused by the position error of UAV *j* to radar *i* implementing jamming is $\Delta {r_{u,\max}}_{k}^{i}$, where the deception distance is $d_{k}^{i}$, the distance from the radar a priori position to the predefined UAV position is ${R_{ru,}}_{k}^{i}$, and the position error of UAV *j* at time *k* is $\Delta {R_{u,}}_{k}^{i}$.

### 3.3. Error Analysis of Time Delay

**Figure 8:**
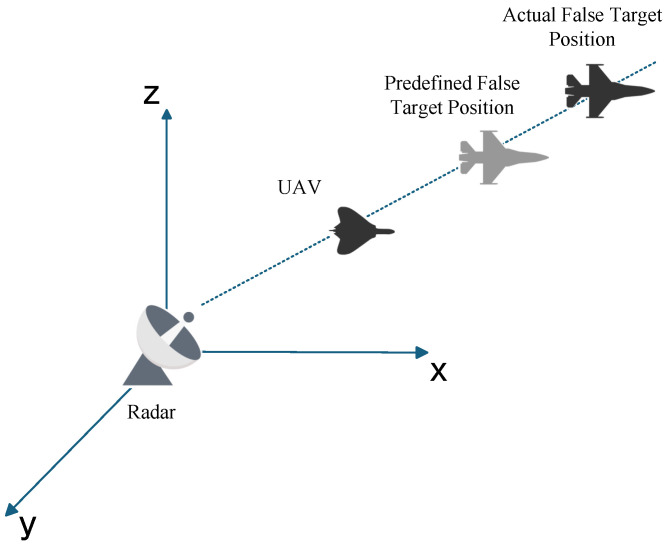
False Target Offset Induced by Time Delay Errors.

The time delay parameter error in radar systems is mainly caused by the combined effect of the inherent characteristics of the system and external environmental factors. The stability of clock sources such as crystal oscillators in the signal generation circuit is insufficient, which will lead to the deviation of the time reference of the interference signal. In addition, the limitation of conversion accuracy and speed in the process of digital-to-analog conversion (DAC) will also cause systematic time delay errors. In terms of the external environment, increased operating temperature or changes in the physical state of the hardware will change the circuit characteristics and then introduce time delay offsets, which may lengthen or shorten the distance of the false target. As shown in Figure 8, time delay errors can also cause false target offset.

Based on radar detection principles, it can be derived that the following relationship exists between time delay error and false target offset:

(20) $$\Delta {r_{\tau ,\max}}_{k}^{i}=\frac{1}{2}c\Delta {\tau_{\max}}_{k}^{j}$$

In Equation (20), at time *k*, the maximum distance of false target offset caused by the delay error of UAV *j* jamming radar *i* is $\Delta {r_{\tau ,\max}}_{k}^{i}$, the maximum delay of UAV *j* is $\Delta {R_{u,}}_{k}^{i}$.

Therefore, under the condition that only time-delay errors exist, the measurement association conditions for two radars are as follows:

(21) $$\frac{1}{2}c\left(\Delta {\tau_{\max}}_{k}^{1}+\Delta {\tau_{\max}}_{k}^{2}\right)\leq \min\left\{\rho_{T_{1}},\rho_{T_{2}}\right\}$$

### 3.4. Error Analysis of Spatial Coupling Synthesis

**Figure 9:**
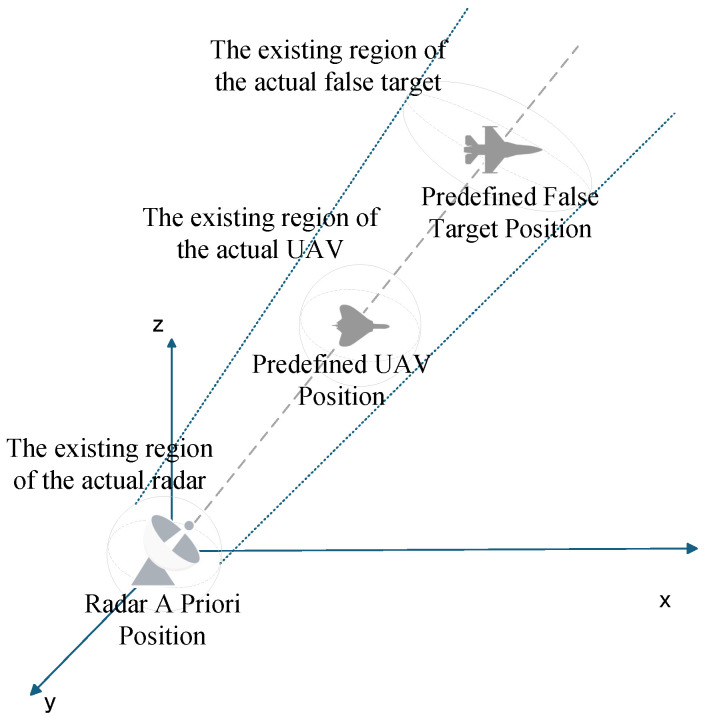
False Target offset Induced by Spatial Coupling Comprehensive Errors.

During the implementation of radar jamming, false targets are subject to the combined effects of multiple error sources. As previously discussed, radar a priori position errors, UAV position errors, and time delay parameter errors each contribute to spatial misalignment of false targets. However, in real-world scenarios (as shown in Figure 9), these errors often act synergistically, necessitating an investigation into whether their impacts are merely additive or involve complex interactions and accumulations that ultimately amplify false target offset.

Assume that the coordinates of the actual radar position are $\left(x_{r^{\prime }},y_{r^{\prime }},z_{r^{\prime }}\right)$, and the coordinates of the predefined UAV position are $\left(x_{u},y_{u},z_{u}\right)$, the coordinates of the actual UAV position are $\left(x_{u^{\prime }},y_{u^{\prime }},z_{u^{\prime }}\right)$, the coordinates of the actual false target position are $\left(x_{j},y_{j},z_{j}\right)$, and the deception distance is d.

(22) $$\left\{\begin{array}{c} x_{j}=x_{u^{\prime }}+\frac{\left(d+\Delta R_{\tau }\right)\left(x_{u^{\prime }}-x_{r^{\prime }}\right)}{\sqrt{{\left(x_{u^{\prime }}-x_{r^{\prime }}\right)}^{2}+{\left(y_{u^{\prime }}-y_{r^{\prime }}\right)}^{2}+{\left(z_{u^{\prime }}-z_{r^{\prime }}\right)}^{2}}}\\ y_{j}=y_{u^{\prime }}+\frac{\left(d+\Delta R_{\tau }\right)\left(y_{u^{\prime }}-y_{r^{\prime }}\right)}{\sqrt{{\left(x_{u^{\prime }}-x_{r^{\prime }}\right)}^{2}+{\left(y_{u^{\prime }}-y_{r^{\prime }}\right)}^{2}+{\left(z_{u^{\prime }}-z_{r^{\prime }}\right)}^{2}}}\\ z_{j}=z_{u^{\prime }}+\frac{\left(d+\Delta R_{\tau }\right)\left(z_{u^{\prime }}-z_{r^{\prime }}\right)}{\sqrt{{\left(x_{u^{\prime }}-x_{r^{\prime }}\right)}^{2}+{\left(y_{u^{\prime }}-y_{r^{\prime }}\right)}^{2}+{\left(z_{u^{\prime }}-z_{r^{\prime }}\right)}^{2}}}\\ x_{r^{\prime }}^{2}+y_{r^{\prime }}^{2}+z_{r^{\prime }}^{2}=\Delta {R_{r}}^{2}\\ {\left(x_{u^{\prime }}-x_{u}\right)}^{2}+{\left(y_{u^{\prime }}-y_{u}\right)}^{2}+{\left(z_{u^{\prime }}-z_{u}\right)}^{2}=\Delta {R_{u}}^{2} \end{array}\right.$$

Assuming the radar priori position error vector is $\Delta R_{r}$ = ($\Delta R_{r},\theta_{1},\varphi_{1}$) and the UAV position error vector is $\Delta R_{u}$ = ($\Delta R_{u},\theta_{2},\varphi_{2}$), $\delta =\cos(\theta_{2}-\theta_{1})$, the probability density functions (PDFs) of the parameters can be obtained as follows:

$$f_{\varphi_{1}}\left(\varphi_{1}\right)=\frac{1}{2}\sin\varphi_{1}\;\left(\varphi_{1}\in \left[0,\pi \right]\right)$$

$$f_{\varphi_{2}}\left(\varphi_{2}\right)=\frac{1}{2}\sin\varphi_{2}\;\left(\varphi_{2}\in \left[0,\pi \right]\right)$$

$$f_{\theta_{1}}\left(\theta_{1}\right)=f_{\theta_{2}}\left(\theta_{2}\right)=\frac{1}{2\pi }\left(\theta_{1}\in \left[0,\pi \right],\theta_{2}\in \left[0,\pi \right]\right)$$

We can derive the distribution function under the condition of comprehensive error as follows:

(23) $$F\left(\Delta r_{s,T}\right)=\frac{1}{8\pi }\int_{0}^{\pi }\int_{0}^{\pi }\int_{0}^{2\pi }\sin\theta_{1}\sin\theta_{2}\cdot 1_{\left\{\Delta r_{s}\leq \Delta r_{s,T}\right\}}\;d\varphi_{1}d\theta_{2}d\theta_{1}$$

Define:

$$D=\sqrt{{\left(\Delta {R_{u,}}_{k}^{j}+\Delta {R_{r,}}_{k}^{i}\right)}^{2}+{{R_{ru,}}_{k}^{i}}^{2}},\;E=d_{k}^{i}+\Delta {R_{\tau ,}}_{k}^{i}$$

Given the complexity of the complete probability density function, an appropriate simplification allows us to derive the maximum false target offset under the influence of spatial coupling comprehensive errors as follows.

(24) $$\Delta {r_{s,\max}}_{k}^{i}=\sqrt{{\left(\Delta {R_{u,}}_{k}^{j}+\frac{(\Delta {R_{u,}}_{k}^{j}+\Delta {R_{r,}}_{k}^{i})E}{D}\right)}^{2}+{\left(\frac{{R_{ru,}}_{k}^{i}E}{D}-d_{k}^{i}\right)}^{2}}$$

In Equation (24), at time *k*, the maximum distance of false target offset caused by the spatial coupling comprehensive errors of UAV *j* jamming radar *i* is $\Delta {r_{s,\max}}_{k}^{i}$, the deception distance is $d_{k}^{i}$, the distance from the radar a priori position to the predefined UAV position is ${R_{ru,}}_{k}^{i}$, the radar a priori position error is $\Delta {R_{r,}}_{k}^{i}$, the position error of UAV *j* is $\Delta {R_{u,}}_{k}^{i}$, and the maximum delay of UAV *j* is $\Delta {R_{u,}}_{k}^{i}$.

The measurement association conditions for two radars under spatial coupling comprehensive errors can be analogously derived from Equation (11), the specific measurement association conditions are as follows.

(25) $$\Delta {r_{s,\max}}_{k}^{1}+\Delta {r_{s,\max}}_{k}^{2}\leq \min\left\{\rho_{T_{1}},\rho_{T_{2}}\right\}$$

Focusing on the scenario with *N* radars, we first analyze the conditions for successfully deceiving Radar $R_{1}$. At time *k*, the measurement association requirements are as follows.

(26) $$\left\{\begin{array}{c} \Delta {r_{s,\max}}_{k}^{1}+\Delta {r_{s,\max}}_{k}^{2}\leq \rho_{T_{1}}\\ \Delta {r_{s,\max}}_{k}^{1}+\Delta {r_{s,\max}}_{k}^{3}\leq \rho_{T_{1}}\\ \ldots \\ \Delta {r_{s,\max}}_{k}^{1}+\Delta {r_{s,\max}}_{k}^{N}\leq \rho_{T_{1}}\\ \Delta {r_{s,\max}}_{k}^{2}+\Delta {r_{s,\max}}_{k}^{3}\leq \rho_{T_{1}}\\ \Delta {r_{s,\max}}_{k}^{2}+\Delta {r_{s,\max}}_{k}^{4}\leq \rho_{T_{1}}\\ \ldots \\ \Delta {r_{s,\max}}_{k}^{2}+\Delta {r_{s,\max}}_{k}^{N}\leq \rho_{T_{1}}\\ \ldots \\ \Delta {r_{s,\max}}_{k}^{N-1}+\Delta {r_{s,\max}}_{k}^{N}\leq \rho_{T_{1}} \end{array}\right.$$

Summing all inequalities in Equation (26) yields the following inequality:

(27) $$\left(N-1\right)\left(\Delta {r_{s,\max}}_{k}^{1}+\Delta {r_{s,\max}}_{k}^{2}+\ldots +\Delta {r_{s,\max}}_{k}^{N}\right)\leq \frac{N(N-1)}{2}\rho_{T_{1}}$$

Simplifying yields the following result:

(28) $$\sum_{i=1}^{N}\Delta r_{s,\max,k}^{i}\leq \frac{N}{2}\rho_{T_{1}}$$

Furthermore, for measurement association among *N* radars, the following conditions must be satisfied:

(29) $$\frac{2}{N}\sum_{i=1}^{N}\Delta r_{s,\max,k}^{i}\leq \min\left\{\rho_{T_{1}},\rho_{T_{2}}\ldots \rho_{T_{N}}\right\}$$

Alternatively, suppose that at time *k*, the maximum false target splitting distances are reordered from smallest to largest as $\left\{\Delta {r_{s,\max}}_{k}^{\left(1\right)},\Delta {r_{s,\max}}_{k}^{\left(2\right)}\ldots \Delta {r_{s,\max}}_{k}^{\left(N\right)}\right\}$.

(30) $$\forall p\in \left\{1,2,\ldots N\right\},\forall q\in \left\{1,2,\ldots N\right\},p\neq q\Rightarrow \Delta {r_{s,\max}}_{k}^{p}+\Delta {r_{s,\max}}_{k}^{q}\leq \rho_{T_{i}}$$

(31) $$\Delta {r_{s,\max}}_{k}^{\left(N\right)}+\Delta {r_{s,\max}}_{k}^{\left(N-1\right)}\leq 2\Delta {r_{s,\max}}_{k}^{\left(N\right)}\leq \rho_{T_{i}}$$

For measurement association among *N* radars, the following conditions must be satisfied:

(32) $$2\Delta {r_{s,\max}}_{k}^{\left(N\right)}\leq \min\left\{\rho_{T_{1}},\rho_{T_{2}}\ldots \rho_{T_{N}}\right\}$$

In summary, the sufficient but not necessary conditions and necessary but not sufficient conditions for measurement association among *N* radars are as follows:

(33) $$\left\{\begin{array}{cc} \frac{2}{N}\sum_{i=1}^{N}\Delta r_{s,\max,k}^{i}\leq \min\left\{\rho_{T_{1}},\rho_{T_{2}}\ldots \rho_{T_{N}}\right\} & \left(\mathrm{Necessary}\;\mathrm{but}\;\mathrm{Not}\;\mathrm{Sufficient}\;\mathrm{Condition}\right)\\ 2\Delta r_{s,\max,k}^{\left(N\right)}\leq \min\left\{\rho_{T_{1}},\rho_{T_{2}},\ldots ,\rho_{T_{N}}\right\} & \left(\mathrm{Sufficient}\;\mathrm{but}\;\mathrm{Not}\;\mathrm{Necessary}\;\mathrm{Condition}\right) \end{array}\right.$$

However, in most cases, the specific magnitude of errors cannot be accurately obtained, and they often follow certain distributions. This paper conducts further research based on the normal distribution. Assuming that the radar priori position error $\Delta R_{r}$, the UAV position error $\Delta R_{u}$, and the time delay error $\Delta R_{\tau }$ follow normal distributions, their probability distributions can be expressed as follows:

$$\left\{\begin{array}{c} \Delta R_{r}∼N\left(\mu_{1},\sigma_{1}^{2}\right)\\ \Delta R_{u}∼N\left(\mu_{2},\sigma_{2}^{2}\right)\\ \Delta R_{\tau }∼N\left(\mu_{3},\sigma_{3}^{2}\right) \end{array}\right.$$

Through analysis, the probability density functions (PDFs) of the parameters can be obtained as follows:

$$f_{|\Delta R_{r}|}\left(r_{1}\right)=\frac{\sqrt{2}}{\sqrt{\pi }k_{1}}\exp\left(-\frac{r_{1}^{2}+\mu_{1}^{2}}{2k_{1}^{2}}\right)\cosh\left(\frac{r_{1}\mu_{1}}{k_{1}^{2}}\right)\;\left(r_{1}>0\right)$$

$$f_{|\Delta R_{u}|}\left(r_{2}\right)=\frac{\sqrt{2}}{\sqrt{\pi }k_{2}}\exp\left(-\frac{r_{2}^{2}+\mu_{2}^{2}}{2k_{2}^{2}}\right)\cosh\left(\frac{r_{2}\mu_{2}}{k_{2}^{2}}\right)\;\left(r_{2}>0\right)$$

$$f_{\delta }\left(\delta \right)=\frac{1}{\pi \sqrt{1-\delta^{2}}}\;\left(\delta \in \left(-1,1\right)\right)$$

$$f_{R_{\tau }}\left(\tau \right)=\frac{1}{\sqrt{2\pi }k_{3}}\exp\left(-\frac{{\left(\tau -\mu_{3}\right)}^{2}}{2k_{3}^{2}}\right)\;\left(R_{\tau }\in R\right)$$

The distribution function of the false target’s deviation distance can then be derived as follows:

(34) $$\begin{array}{c} F\left(\Delta r_{s^{\prime },T}\right)=\int_{0}^{\infty }\int_{0}^{\infty }\int_{0}^{\pi }\int_{0}^{\pi }\int_{-1}^{1}\int_{-\infty }^{\infty }1_{\left\{\Delta r_{s^{\prime }}\leq {\Delta r_{s^{\prime },T}}^{2}\right\}}f_{|\Delta R_{r}|}\left(r_{1}\right)f_{|\Delta R_{u}|}\left(r_{2}\right)\\ f_{\varphi_{1}}\left(\varphi_{1}\right)f_{\varphi_{2}}\left(\varphi_{2}\right)f_{\delta }\left(\delta \right)f_{\tau }\left(\tau \right)\;d\tau d\delta d\varphi_{2}d\varphi_{1}dr_{2}dr_{1} \end{array}$$

Combining with Equation (9), the information entropy of false targets can be obtained as follows:

(35) $$H=-\sum_{j=1}^{K}p_{j}\log_{2}p_{j}=-\sum_{i=1}^{N}F_{i}\left(\Delta r_{s^{\prime },T}\right)\log_{2}F_{i}\left(\Delta r_{s^{\prime },T}\right)$$

Therefore, with a 95% significance detection threshold, the jamming effectiveness must satisfy the following condition:

(36) $$H<-\sum_{i=1}^{N}0.95\log_{2}0.95$$

## 4. Solution Techniques

Through geometric analysis and algebraic manipulation, the previous section derived sufficient but not necessary conditions for measurement association among *N* radars under spatial coupling errors. When these conditions are not met in practical scenarios, it is imperative to develop practical and effective error compensation schemes to ensure successful jamming implementation. For track deception, the primary research focuses on the target radar, UAV, and jamming payload. Given that the target radar is a noncooperative entity, this paper proposes two compensation schemes targeting UAVs and jamming payloads.

### 4.1. Zonal Track Compensation Method

**Figure 10:**
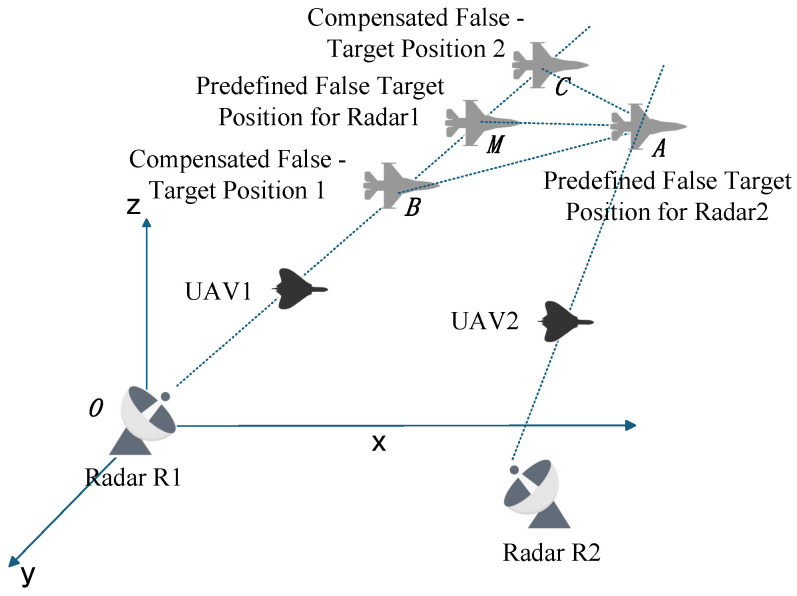
Schematic of the Zonal Track Compensation Method.

The zonal track compensation method, as the name implies, this method ultimately forms a target false track by creating a zonal track. The underlying principle involves generating multiple additional false targets around the originally induced false target, thereby increasing the probability of measurement association at each time step and ensuring the production of point traces suitable for track formation. As shown in Figure 10, *M* and *A* represent the false target points generated by Radar $R_{1}$ and Radar $R_{2}$, respectively.

Taking radar $R_{1}$ as an example, where α is a constant greater than zero, the measurement association conditions fail to hold under the following circumstances:

(37) $$\left|\rho_{k}^{1}-\rho_{k}^{21\prime }\right|=\rho_{T_{1}}+\alpha$$

At this time, two additional false targets *B* and *C* are generated at a distance β from the original false target *M*, where |BM|=|CM|=β. The value of β is defined as follows:

(38) $$\left\{\begin{array}{c} \beta =\alpha +k\rho_{T_{1}}\;\mathrm{when}\;\rho_{T_{1}}\leq \alpha \\ \beta =\rho_{T_{1}}+k\alpha \;\mathrm{when}\;\rho_{T_{1}}>\alpha . \end{array}\right.$$

In Equation (38), k∈(0,1). Consequently, the following scenarios can be derived:

(39) $$\min\left|\rho_{k}^{1}\pm \beta -\rho_{k}^{21\prime }\right|<\rho_{T_{1}}$$

From a geometric perspective, when ΔABC is an obtuse triangle with AM as the median to side BC, if ∠ACB is obtuse, then |AC|<|AM|. This result can be derived as follows:

(40) $$\left\{\begin{array}{c} \left|\rho_{k}^{1}-\rho_{k}^{21\prime }\right|<\left|AC\right|<\left|AM\right|\\ \left|\rho_{k}^{2}-\rho_{k}^{12\prime }\right|<\left|AC\right|<\left|AM\right| \end{array}\right.$$

Comparison with Equation (11) reveals that compensation reduces the Euclidean distance between false target splitting points, thereby reducing the required size of the association gate and increasing the measurement association probability.

Generalizing to the case of *N* radars, from an algebraic perspective, the zonal track compensation Method can be interpreted as systematically modifying time delay errors. By taking the derivative of $\Delta R_{\tau ,k}^{i}$ in Equation (24), the increasing and decreasing intervals are derived as Equation (41) and Equation (42), respectively:

(41) $$\Delta {R_{\tau ,}}_{k}^{i}>-d_{k}^{i}-\frac{\Delta {R_{u,}}_{k}^{j}\left(\Delta {R_{u,}}_{k}^{j}+\Delta {R_{r,}}_{k}^{i}\right)-{R_{ru,}}_{k}^{i}d_{k}^{i}}{\sqrt{{\left(\Delta {R_{u,}}_{k}^{j}+\Delta {R_{r,}}_{k}^{i}\right)}^{2}+{{R_{ru,}}_{k}^{i}}^{2}}}$$

(42) $$\Delta {R_{\tau ,}}_{k}^{i}<-d_{k}^{i}-\frac{\Delta {R_{u,}}_{k}^{j}\left(\Delta {R_{u,}}_{k}^{j}+\Delta {R_{r,}}_{k}^{i}\right)-{R_{ru,}}_{k}^{i}d_{k}^{i}}{\sqrt{{\left(\Delta {R_{u,}}_{k}^{j}+\Delta {R_{r,}}_{k}^{i}\right)}^{2}+{{R_{ru,}}_{k}^{i}}^{2}}}$$

From Equations (41) and (42), it can be derived that $\Delta r_{s,\max,k}^{i}$ attains a minimum value, which is given by:

(43) $${\left(\Delta {r_{s,\max}}_{k}^{i}\right)}_{\min}=\frac{{R_{ru,}}_{k}^{i}\Delta {R_{u,}}_{k}^{j}+d_{k}^{i}\left(\Delta {R_{u,}}_{k}^{j}+\Delta {R_{r,}}_{k}^{i}\right)}{\sqrt{{\left(\Delta {R_{u,}}_{k}^{j}+\Delta {R_{r,}}_{k}^{i}\right)}^{2}+{{R_{ru,}}_{k}^{i}}^{2}}}$$

When a minimum is achieved, errors in arbitrary directions can be mitigated; however, the compensation effectiveness varies with error orientation. Therefore, given prior information about the deception distance $d_{k}^{i}$, the distance from the radar’s prior position to the pre-positioned UAV $R_{ru,k}^{i}$, the radar’s prior position error $\Delta R_{r,k}^{i}$, the time delay error $\Delta R_{\tau ,k}^{i}$, and the UAV position error $\Delta R_{u,k}^{j}$, an appropriate β value can be determined to minimize $\Delta r_{s,\max,k}^{i}$, thereby reducing the impact of comprehensive errors. Additionally, empirical formulas derived from engineering practice can be developed for convenient computation.

### 4.2. Formation Jamming Compensation Method

**Figure 11:**
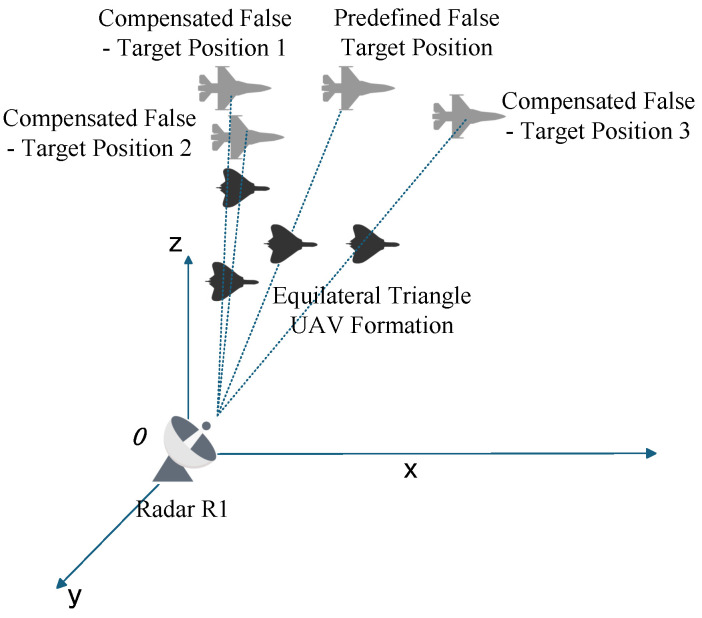
Schematic of the Formation Jamming Compensation Method.

The formation jamming compensation method evolves from single—UAV jamming against a single radar at an initial time step to UAV formation jamming against the same radar. As shown in Figure 11, a four—UAV formation is typically employed in an equilateral triangle configuration, where the geometric center coincides with the original single—UAV jamming position. The distance from each of the three vertex UAVs to the center is $D_{u}$.

When the main lobe of the target radar illuminates the UAV at the geometric center, multiple UAVs simultaneously generate false targets along the line connecting each radar to its corresponding UAV. To rigorously analyze this from an algebraic perspective, we take the derivative of $\Delta R_{u,k}^{i}$ in Equation (24) and derive the increasing interval as follows.

(44) $$\Delta {R_{u,}}_{k}^{j}>-\frac{\left(\Delta {R_{u,}}_{k}^{j}+\Delta {R_{r,}}_{k}^{i}\right)\left(d_{k}^{i}+\Delta {R_{r,}}_{k}^{i}\right)}{\sqrt{{\left(\Delta {R_{u,}}_{k}^{j}+\Delta {R_{r,}}_{k}^{i}\right)}^{2}+{{R_{ru,}}_{k}^{i}}^{2}}}$$

**Figure 12:**
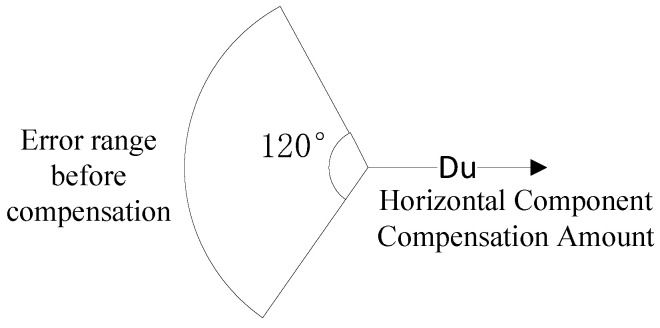
Simplified Horizontal Compensation Model for UAV Formations.

Since the right-hand side of the inequality is always negative, $\Delta r_{s,\max,k}^{i}$ is a monotonically increasing function of $\Delta R_{u,k}^{i}$ over (0,+∞). Therefore, the basic compensation strategy is to reduce UAV position errors. Let the pitch and azimuth angles of the UAV position error $\Delta R_{u}$ be φ and θ, respectively, which can be represented in vector form as $\Delta R_{u}\left(\cos\varphi \cos\theta ,\cos\varphi \sin\theta ,\sin\varphi \right)$. From another perspective, the three newly added UAVs introduce errors with magnitude $D_{u}$, pitch angle $0^{\circ }$, and azimuth angles $0^{\circ }$, $120^{\circ }$, and $240^{\circ }$ relative to the original $\Delta R_{u}$. Due to the equilateral triangle formation, Figure 12 illustrates the simplified model leveraging geometric symmetry.

Consequently, the compensated error is derived as follows:

(45) $$\left\{\begin{array}{c} \Delta {R_{u}^{\prime }}_{x}=D_{u}+\Delta R_{u}\cos\varphi \cos\theta \\ \Delta {R_{u}^{\prime }}_{y}=\Delta R_{u}\cos\varphi \sin\theta \\ \Delta {R_{u}^{\prime }}_{z}=\Delta R_{u}\sin\varphi \end{array}\right.$$

From the above equation, it can be obtained that this compensation method mainly compensates for the horizontal component of the UAV position error. To make the UAV position error effectively compensated, the magnitude of the compensated UAV position error $\Delta R_{u}^{\prime }$ should be smaller than the original $\Delta R_{u}$. After simplification, the magnitude of $\Delta R_{u\;x}^{\prime }$ can be obtained to be smaller than the original $\Delta R_{u\;x}$, as follows:

(46) $$\left\{\begin{array}{l} \left|D_{u}+\Delta R_{u}\cos\varphi \cos\theta \right|<-\Delta R_{u}\cos\varphi \cos\theta \\ -\frac{\pi }{2}\leq \varphi \leq \frac{\pi }{2}\\ \frac{2\pi }{3}\leq \theta \leq \frac{4\pi }{3} \end{array}\right.$$

Since the horizontal component compensation effectiveness degrades when φ approaches the boundary (where the value approaches zero), to ensure the compensation scheme remains effective under most conditions, we assume $D_{u}=q\Delta R_{u}\left(q>0\right)$ and rederive the valid range of φ using Equation (47) as follows.

(47) $$-\arccos\frac{-q}{2\cos\theta }\leq \varphi \leq \arccos\frac{-q}{2\cos\theta }$$

From Equation (47), it can be derived that *q* satisfies the following condition:

(48) $$\arccos q\leq \arccos\frac{-q}{2\cos\theta }\leq \arccos\frac{q}{2}$$

Therefore, the range of φ is as follows.

(49) −arccosq≤φ≤arccosq

To ensure the compensation probability exceeds 70%, the following formula is derived:

(50) $$\arccos q>\frac{7}{20}\pi$$

And then, we derive that $q<\cos\frac{7}{20}\pi$. Specifically, when the UAV position error $\Delta R_{u}$ is known, using an equilateral triangle formation with vertex UAVs positioned at $D_{u}\in \left(0,\cos\frac{7}{20}\pi \Delta R_{u}\right)$ from the center achieves compensation effectiveness in over 70% of scenarios.

The previously analyzed compensation implementation using a triangular formation represents a trade-off between compensation effectiveness and jamming resource utilization. However, when prioritizing compensation performance under sufficient jamming resources, the formation jamming strategy can be generalized to a regular n-gon configuration. For a given UAV position error *R* and a required compensation probability exceeding m%, the following condition is derived:

(51) $$\left\{\begin{array}{c} \left|D_{u}+\Delta R_{u}\cos\varphi \cos\theta \right|<-\Delta R_{u}\cos\varphi \cos\theta \\ -\frac{m}{200}\pi \leq \varphi \leq \frac{m}{200}\pi \\ \pi -\frac{\pi }{n}\leq \theta \leq \pi +\frac{\pi }{n} \end{array}\right.$$

Similarly, it can be derived that *q* must satisfy the following condition under this constraint:

(52) $$\arccos\frac{q}{2\cos\frac{\pi }{n}}>\frac{m}{200}\pi$$

(53) $$q<2\cos\frac{\pi }{n}\cos\frac{m}{200}\pi$$

In summary, when using the equilateral *n*-sided formation jamming for compensation, a sufficient but not necessary condition for the compensation probability to reach m% is $D_{u}<2\cos\frac{\pi }{n}\cos\frac{m}{200}\pi q$. Moreover, as can be seen from Equation (53), the larger the value of *n*, the larger the range of values for *q*; conversely, the larger the value of *m*, the smaller the range of values for *q*. Although this paper has established a model and derived the setting conditions for the jamming formation, in engineering practice, due to the minimum distance constraint between drones, the compensation probability can be appropriately reduced. Alternatively, the formation jamming can be scaled down to the arraying of jammer antennas to compensate for the position errors of drones.

Algorithm 1 summarizes the error-aware compensation workflow applied at each radar scan step: it evaluates the upper-bound offset induced by coupled uncertainties and conditionally applies zonal or formation compensation to maintain measurement consistency and coherent false-target tracks.
**Algorithm 1:** Error-Aware Compensation Workflow**Input:** estimated error bounds $\left\{\Delta R_{r},\Delta R_{u},\Delta \tau \right\}$, association threshold $\rho_{T}$**Output:** adjusted compensation parameters $\left\{\Delta R_{\tau }^{\prime },D_{u}\right\}$1 **for** each radar scan step *k* **do**2    compute upper-bound offset $\Delta r_{\max}$ using Equation (24)3    **if** $\Delta r_{\max}>\rho_{T}$ **then**4       adjust $\Delta R_{\tau }$ heuristically per Equations (41)–(43)5       recompute $\Delta r_{\max}$6    **end if**7    **if** $\Delta r_{\max}>\rho_{T}$ **then**8       adjust UAV geometry: set $D_{u}=q\cdot \Delta R_{u}$ with *q* satisfying Equation (53)9    **end if**10   record compensation parameters for step *k*11 **end for**

## 5. Simulation and Results

### 5.1. Model Validation

**Figure 13:**
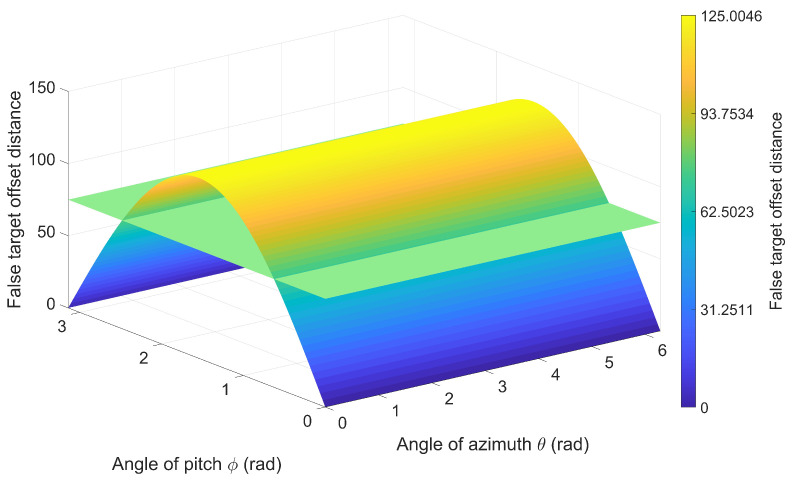
False Target Offset Induced by A Priori Position Errors of Multidirectional Radars.

**Figure 14:**
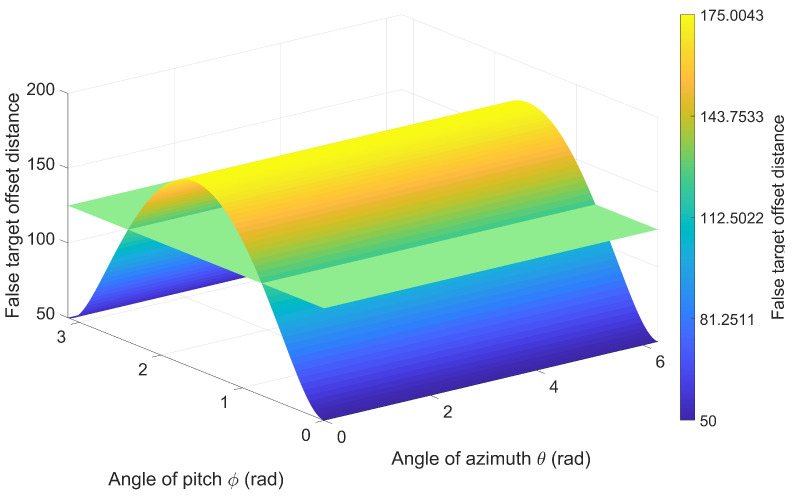
False Target Offset Induced by Position Errors of Multidirectional UAVs.

**Figure 15:**
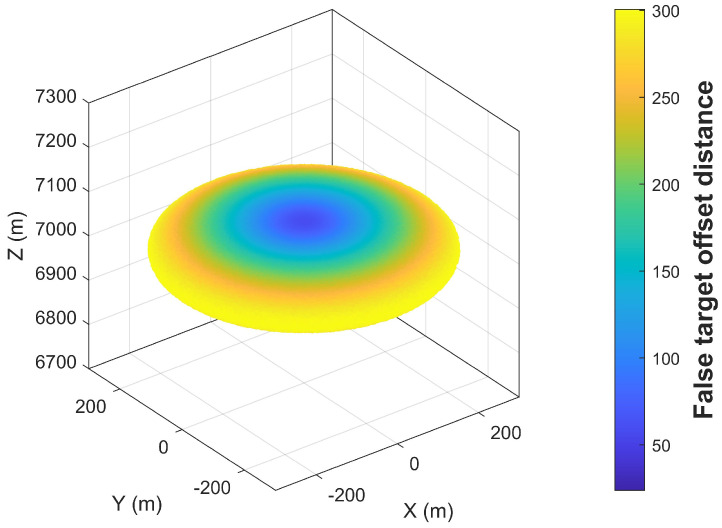
False Target Offset Induced by Multidirectional Combined Errors.

**Figure 16:**
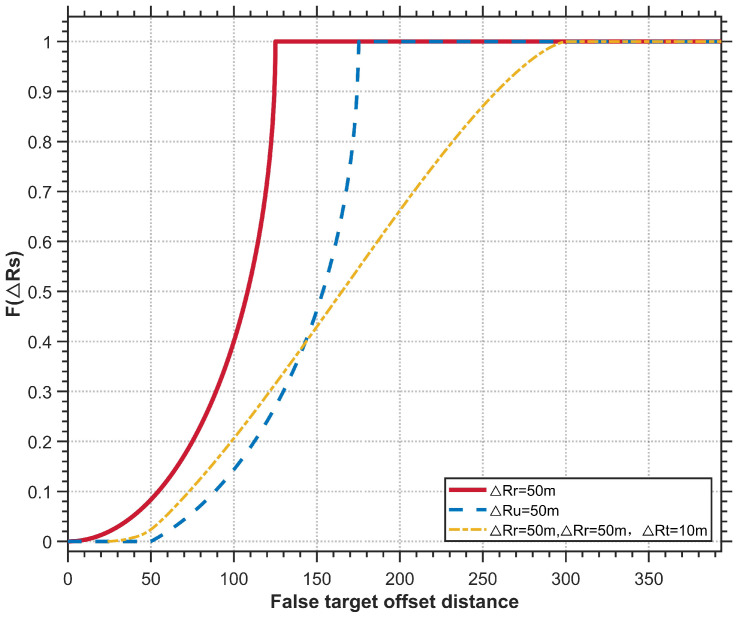
False Target Offset Euclidean Distance Distribution Curve.

Section 5.1 was designed to investigate the effects of multidirectional radar a priori position errors and UAV position errors on false target offset in a scenario where a single UAV jams a single radar. The experimental conditions are presented in Table 1. Figure 13 and Figure 14 illustrate the false target offset caused by individual error sources, while Figure 15 demonstrates the cumulative effects of interacting combined errors, and Figure 16 illustrates the distribution functions under single-error conditions and comprehensive- error conditions.

As presented in Figure 13 and Figure 14, the *x*-axis represents the azimuth angle of the error, the *y*-axis represents the elevation angle of the error, the *z*-axis represents the false target offset distance, and the color bar represents the magnitude of offset distance. As shown in the results, the surface represents the actual offset, while the plane denotes the estimated offset obtained from Equations (10) and (16). The estimated offset can be used to represent the actual offset in most cases. Additionally, comparing the maximum value of the actual offset with parameter $\Delta r_{s,\max}$ in Table 1 validates Equations (15) and (19).

Due to the four-dimensional nature of angular variables in combined errors, which complicates visualization, Figure 15 presents only the false target distribution under the experimental conditions. Analysis of experimental conditions and results reveals that combined errors do not merely represent linear superposition of individual error sources but instead induce false target offset through complex interactions and cumulative effects. This also validates Equation (24). Figure 16 illustrates the distribution functions of the false target’s deviation distance under three different error scenarios, which validates Equations (14), (18) and (34) derived in Section 3. Collectively, Section 5.1 confirms the correctness of the error model developed in this paper.

### 5.2. Compensation Evaluation

**Figure 17:**
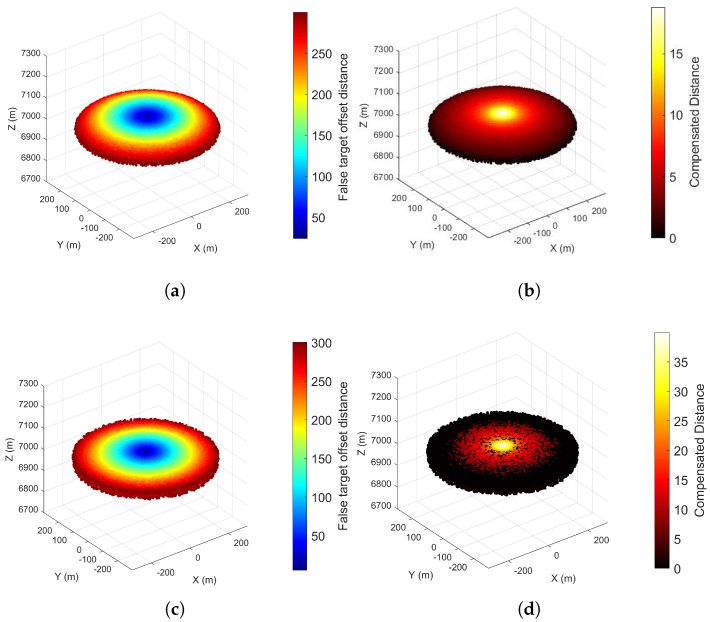
Compensatory Performance of Zonal Trajectory Compensation Method for Offset Distance (**a**) $R_{\tau }$**= 8.78 m**, False Target Offset Distribution. (**b**) $R_{\tau }$**= 8.78 m**, Compensated Distance. (**c**) $R_{\tau }$**= 30 m**, False Target Offset Distribution. (**d**) $R_{\tau }$ **= 30 m**, Compensated Distance.

**Figure 18:**
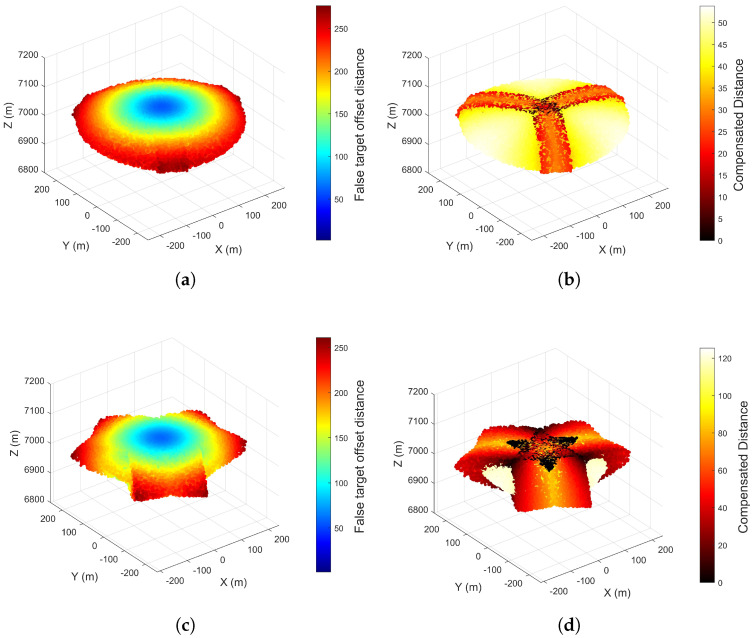
Compensatory Performance of Formation Jamming Compensation Method for Offset Distance (**a**) **q = 0.3**, False Target Offset Distribution. (**b**) **q = 0.3**, Compensated Distance. (**c**) **q = 0.7**, False Target Offset Distribution. (**d**) **q = 0.7**, Compensated Distance.

Section 5.2 was designed to validate compensation schemes for a single UAV jamming a single radar. Pre-compensation results can be found in Figure 15 of Section 5.1. Under identical error conditions, Section 5.2 focuses on comparing results across different compensation parameters and schemes. The final experimental results are presented in Figure 17 and Figure 18.

Figure 17 compares two scenarios with different time delay compensation distances. The compensation parameters in Figure 17a,b are derived from Equations (41) and (42). By comparing the maximum offset distances in Figure 17a,c and analyzing color bar differences in Figure 17b,d, it is validated that the optimal compensation parameters minimize the maximum false target offset while maintaining effectiveness across most scenarios. However, comparisons of maximum compensated distances in Figure 17b,d reveal that non-optimal parameters achieve better compensation along the line connecting the radar a priori position and the UAV’s pre-determined position.

Figure 18 compares two scenarios with different values of the formation distance-to-UAV position error ratio q. The spatial symmetry of the compensation scheme is validated through analysis of the four subfigures. The formation compensation parameters in Figure 18a,b satisfy Equation (50). Comparison of Figure 18a,c and color bar analysis reveals that compensated parameters meeting Equation (50) conditions exhibit larger average false target offsets. Conversely, Figure 18b,d comparisons show that parameters satisfying Equation (50) achieve higher compensation probability but lower compensation performance compared to non-compliant parameters. This pattern extends to equilateral n-sided formations. Therefore, practical compensation implementations require balancing compensation probability and performance and setting appropriate parameters according to specific needs.

**Figure 19:**
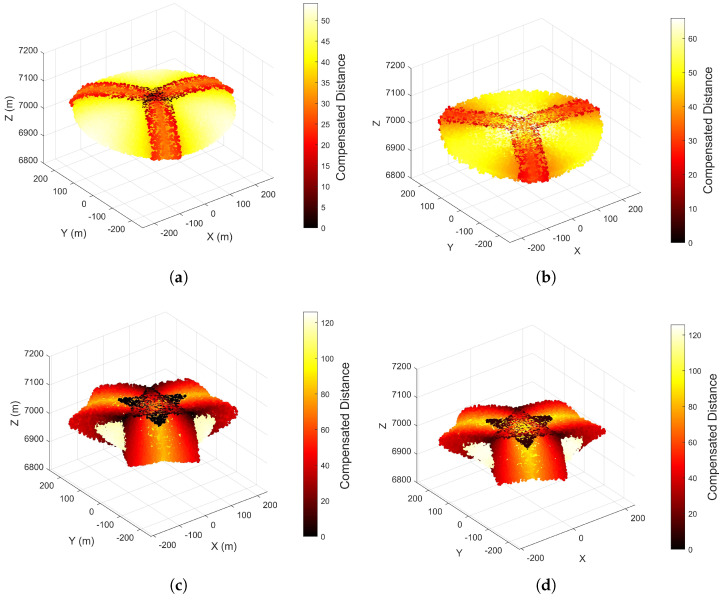
Compensatory Performance of Combined Compensation Method for Offset Distance (**a**) **q = 0.3**, **$R_{\tau }$ = 20 m**, False Target Offset Distribution. (**b**) **q = 0.3**, **$R_{\tau }$ = 30 m**, Compensated Distance. (**c**) **q = 0.7**, **$R_{\tau }$ = 20 m**, False Target Offset Distribution. (**d**) **q = 0.7**, **$R_{\tau }$ = 8.78 m**, Compensated Distance.

Based on the analysis of Figure 17 and Figure 18, it is proposed to implement a combined application of the two compensation schemes, which are mutually complementary. A joint experiment was conducted under the experimental conditions of Figure 17 and Figure 18, with results presented in Figure 19.

As presented in Figure 19, the formation compensation method is used as the foundation, and the zonal trajectory compensation method is implemented simultaneously. As analyzed earlier, when either compensation scheme is applied alone, it is difficult to balance compensation performance and probability. For Figure 19a, which emphasizes compensation probability, by incorporating zonal trajectory compensation parameters that focus on compensation performance, Figure 19b is obtained. Similarly, for Figure 19c, which emphasizes compensation performance, by incorporating zonal trajectory compensation parameters that focus on compensation probability, Figure 19d is achieved. By comparing the central regions of the two figures with the same formation compensation parameters, it can be observed that the central area in the combined compensation results has a lighter color and fewer dark spots. This indicates that the combined compensation method has a favorable effect on balancing compensation performance and probability.

### 5.3. Multi-UAV Performance

**Figure 20:**
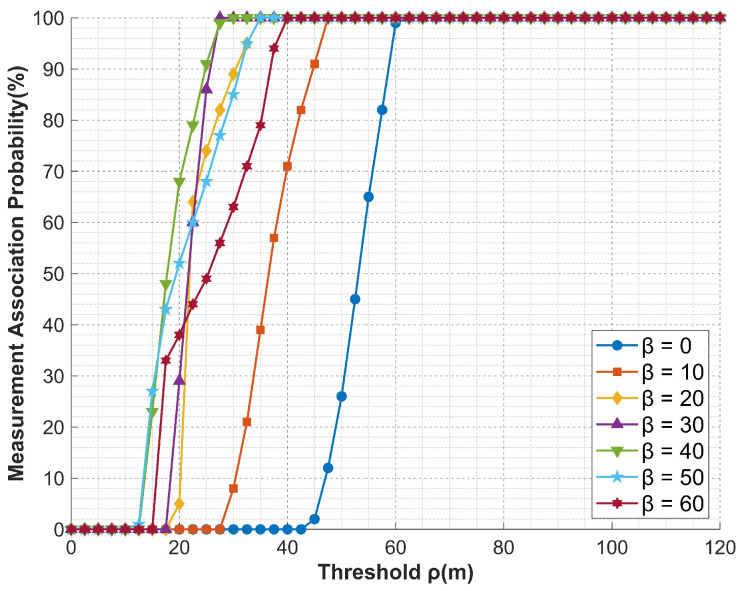
Compensation effect curve of the zonal track compensation method.

**Figure 21:**
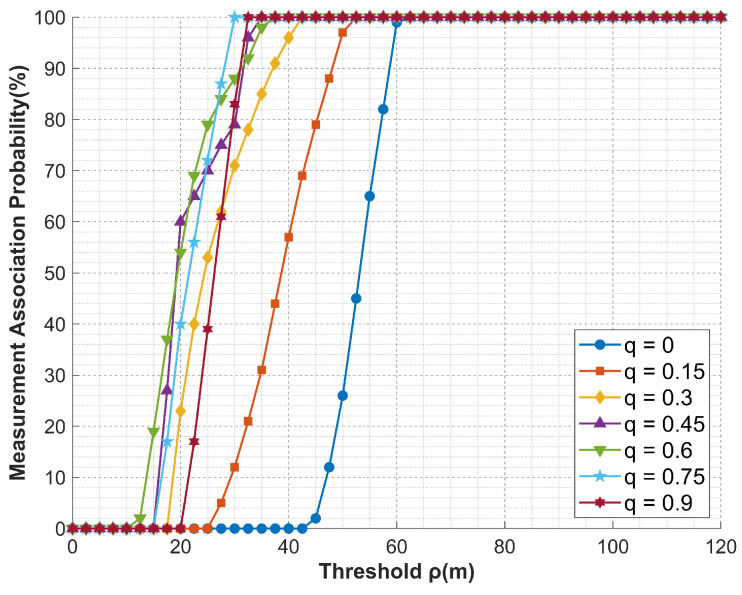
Compensation effect curve of the formation jamming compensation method.

**Figure 22:**
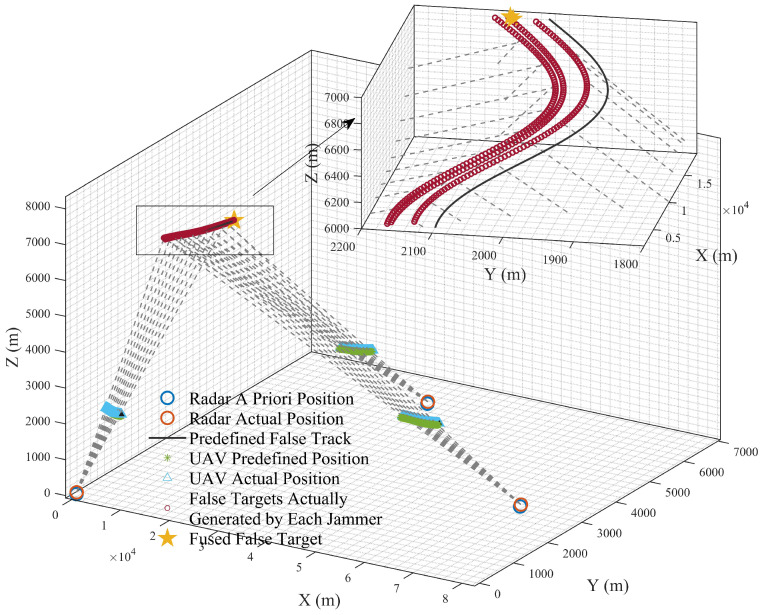
Uncompensated track deception overall scenario.

**Figure 23:**
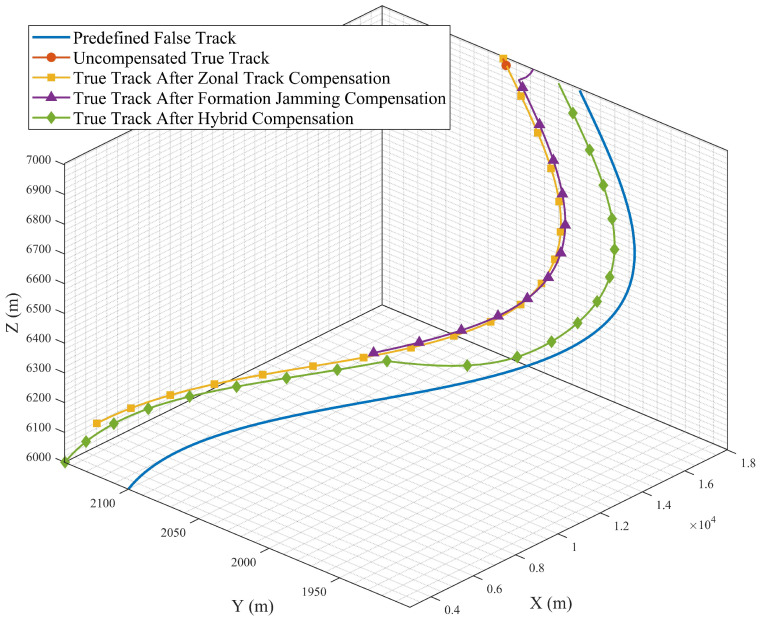
Tracks formed after filtering under different conditions.

Section 5.3 conducts simulation analysis on false target measurement association for cooperative track deception jamming of multi-UAVs against networked radars under error conditions. The simulated false trajectory is defined by Equation (54), and the basic experimental conditions are presented in Table 2 and Table 3, which validates the effectiveness of compensation schemes in practical scenarios. The experimental results are shown in Figure 20 and Figure 21. The false track effects displayed under specific scenarios and after filtering are presented in Figure 22 and Figure 23.

(54) $$\left\{\begin{array}{c} x(t)=3000+150t;\\ y(t)=100\cos(0.015\pi (t+1))+2000;\\ z(t)=10t+6000; \end{array}\right.$$

From the experimental results, it can be observed that both compensation strategies exhibit favorable compensation effects on multi-UAV cooperative track deception jamming. Under the simulation conditions, taking 100% measurement association probability as an example, the zonal trajectory compensation method reduces the threshold requirement from 70 to a maximum of 27.5, while the formation jamming compensation method achieves a maximum reduction to 30. Additionally, under the same conditions, the compensation effect of the compensation parameters shows a phenomenon of increasing first and then decreasing as the parameters increase, which is consistent with the derivation in Section 4.

Figure 22 demonstrates the simulation results of the overall scenario where multiple UAVs conduct coordinated track deception against a networked radar under error conditions. The experimental results show that the presence of errors causes a spatially coupled false track to deviate from its predefined trajectory and split into three independent tracks. Under the experimental conditions, the measurement association criteria are satisfied only at one time instant, leading to the degradation of tracks into point traces and significantly impairing the jamming effect.

Figure 23 respectively shows the false tracks finally formed after data processing under four scenarios. As shown in the results, the compensation schemes exhibit superior compensation performance, and the tracks formed by the joint application of the two schemes are closer to the predefined tracks. To more intuitively demonstrate the effectiveness and robustness of the compensation scheme proposed in this paper, we set a=10 m and $D_{u}=20$ m, and validate the scheme under different error conditions (where all errors follow normal distributions). The means of $R_{r}$ and $R_{u}$ are varied, with their standard deviations both set to 10 m; the mean of $R_{\tau }$ is 10 m, and its standard deviation is 5 m. Under these experimental conditions, 10,000 Monte Carlo simulations are conducted, and the average value is taken as the deception success probability. The final experimental results are shown in Table 4, where Z denotes the strip-track compensation method and F denotes the formation jamming compensation method.

As can be seen from the experimental results, even without adaptive adjustment of compensation parameters, the scheme can still maintain good compensation performance under different error conditions. Meanwhile, under various error conditions, the combined effect of the two compensation schemes outperforms that of a single compensation scheme. Therefore, the compensation scheme proposed in this paper exhibits both robustness and effectiveness.

## 6. Conclusions and Future Work

This study proposed an error-aware deception framework that explicitly models radar- position, UAV-navigation, and time-delay uncertainties—and, crucially, captures their spatial coupling. By analyzing how each error source inflates false-target splitting, we derived closed-form conditions under which deceptive measurements remain mutually consistent across radars. These theoretical results were then translated into two practical compensation schemes:zonal trajectory compensation, which inserts auxiliary false targets to form a dense deception belt, and formation-based compensation, which reshapes the swarm geometry to suppress horizontal error components.

Without compensation, the correlation-gate radius had to be about 60 m to maintain even modest track coherence. This large gate was required because coupled position and timing errors substantially displaced the false targets. The proposed zonal-trajectory compensation reduced the required gate to 27.5 m and markedly improved false-target alignment across multiple radars. Likewise, the formation-based compensation lowered the gate threshold to approximately 30 m and produced stable track structures across diverse error orientations. Simulations further showed that combining both strategies provided the best trade-off between compensation effectiveness and robustness. Under representative simulation settings involving radar-position errors of 50 m and time-delay offsets of 10 m, the combined compensation method maintained measurement association across all evaluated time steps. These findings indicate that the joint compensation scheme markedly enhances the reliability and spatial coherence of multi-UAV deception under realistic uncertainties, thereby reducing the gap between theory and practice.

Despite these gains, several limitations remain. The present model omits dynamic radar-cross-section variations, Doppler-velocity coupling, and channel-specific electromagnetic distortions. Furthermore, the compensation logic is purely geometric; questions of power allocation, waveform design, and cognitive electronic-warfare adaptation are left unaddressed. Finally, all evaluations were performed in simulation, so real-time feedback constraints on UAV manoeuvring were not fully captured.

Future research will therefore move in four directions. First, we will extend the homology test to incorporate RCS variability and Doppler misregistration, enabling deception that is robust against velocity-gated fusion filters. Second, an adaptive compensation controller will be developed by embedding an online Kalman estimator that updates error bounds and tunes compensation gains during flight, thus enhancing resilience to wind-induced perturbations and clock drifts. Third, hardware-in-the-loop trials—with a software-defined-radio swarm platform—will be conducted to validate latency, power consumption, and spectral-mask compliance under dense electronic-warfare conditions. Finally, we aim to generalize the framework to cross-domain scenarios in which RF decoys are synchronized with infrared flares, allowing a coordinated attack on multi-sensor fusion radars. Collectively, these efforts will advance the proposed methodology from high-fidelity simulation toward field-deployable multi-UAV deception technology.

## Figures and Tables

**Table 1:** False Target Offset Simulation: Experimental Condition Configuration.

Figure	$R_{r}$ (m)	$R_{u}$ (m)	$R_{\tau }$ (m)	$\Delta r_{s,\max}$ (m)
Figure 13	50	0	0	125
Figure 14	0	50	0	175
Figure 15	50	50	10	300.15

**Table 2:** Radar A Priori Position and Associated Error Parameter Conditions.

Symbol	Coordinate (km)	$\Delta R_{r}$ (m)	Azimuth Angle of $R_{r}$ (°)	Elevation Angle of $R_{r}$ (°)
Radar $R_{1}$	(0,0,0)	50	30	80
Radar $R_{2}$	(30,6,0)	50	45	70
Radar $R_{3}$	(70,3,0)	50	60	60

**Table 3:** UAV-Associated Error Parameter Conditions.

Symbol	$\Delta R_{\tau }$ (m)	$\Delta R_{u}$ (m)	Azimuth Angle of $R_{u}$ (°)	Elevation Angle of $R_{u}$ (°)
${UAV}_{1}$	10	50	30	60
${UAV}_{2}$	10	50	45	20
${UAV}_{3}$	10	50	60	30

**Table 4:** Comparison Table of Compensation Effects with Given Compensation Parameters Under Different Error Conditions.

$\Delta R_{r}$ = $\Delta R_{u}$ (m)	Uncompensated Fusion Success Rate (%)	The Fusion Success Rate of Z (%)	The Fusion Success Rate of F (%)	The Fusion Success Rate of Z + F (%)
40	41	100	96	100
50	2	91	60	100
60	0	57	41	90
70	0	19	29	73

## Data Availability

Data are available from the corresponding author upon request.
